# Pressure in the External Auditory Meatus—A New Treatment

**Published:** 1882-06

**Authors:** George H. Noble

**Affiliations:** Atlanta, Ga.


### PRESSURE IN THE EXTERNAL AUDITORY MEATUS—A NEW TREATMENT.

By GEO. H. NOBLE, M.D., Atlanta, Ga.

Reasoning from the good effects of the vaginal tampon of my professional associate, Prof. Taliaferro, I determined to try the efficacy of a similar treatment in the ear; especially in such cases as those when the meatus auditorius externus is so greatly swollen that a satisfactory examination cannot be made, or where the canal is greatly reduced in size or calibre. This symptom or condition, then, is to be treated rather than the disease, in as much as it is beyond our reach in many of these cases. After which, this tumefied condition having been removed, we combat the disease with such means as are ordinarily at hand. Even in cases where the canal is not much reduced, we find this treatment to be of the greatest assistance in relieving the general engorgement. I do not confine it to any one disease, but apply it in all swollen conditions of the external auditory canal—except where there is the so-called furuncle. It is not necessary to mention the diseases in which we meet with this tumefaction, as any one at all familiar with the diseases of the ear well knows in what troubles we find it most. The object is to remove this swelling, engorgement or obstruction, and this we do by nicely and evenly, yet firmly placing in the ear some soft, yielding substance, which, by its elasticity, keeps up a continuous pressure on the surrounding parts, supports and controls the circulation, and causes the removal of all surplus or foreign material or matter. We take quite a number of small pledgets of cotton—varying in size according to the degree of encroachment of the swelling upon the canal, or as large as can be easily introduced deeply into the meatus—and around each of them tie a thread, that we may manage them the more easily. Saturate the first few pledgets in glycerine, or such medicated solutions as may be deemed best to be used. The glycerine is about as good as anything, and should be used in connection with the other medicaments, if not alone. Then, with a small probe or forceps, pass the cotton deeply in and down quite near the site of the drum membrane, taking care not to impinge upon that membrane or the ossicles if they be in tact or partly so. Then follow with a second and a third piece, etc Then pull upon the threads to tighten the cotton and force a plug in the deep portion of the meatus to pack the meatus against. Keep moderate tension upon these threads as you pack the ear, until completely filled. After the first few pieces, the cotton should be put in dry in order that we may get the benefits of its elasticity, which indeed is a great point in this treatment. As the plugs or pledgets are put in, the threads should be cut successively shorter and shorter, so that the last may be known by the shortest thread, etc. In removing the "tampon" we first pull upon the last or shortest thread, thereby removing the last introduced plug of cotton, and then the next shortest thread, and so on until all have been withdrawn. If there be not much suppuration this application may be left for two days or more, but if the discharge be great or offensive the daily removal and re-application will be necessary, which latter I prefer. I find glycerine; glycerine, iodoform and balsam Peru; glycerine and nitrate of silver; tano-glycerine and boracic acid to be of great service in this treatment. Sometimes an anodyne may be added to assuage the unpleasantness often present.

The success depends mostly upon the tension with which the cotton is applied and thorough cleansing when removed. A few days will generally suffice to remove this swollen and inflamed condition of the ear, and then the more ordinary plan of treatment may be pursued for the cure of the disease proper. The great object is to get the proper degree of elastic pressure, and for that puspose I suppose any soft, elastic substance will do. Probably a rubber tube, so that the secretions may escape, or the tube with cotton packed around it. But this I have not tried, and really do not know whether the tube will be of much avail or not, but the cotton has brought about the most happy results.

